# Malaria and anemia prevention in pregnant women of rural Burkina Faso

**DOI:** 10.1186/1471-2393-4-18

**Published:** 2004-08-27

**Authors:** Caroline Miaffo, Florent Some, Bocar Kouyate, Albrecht Jahn, Olaf Mueller

**Affiliations:** 1 Ministry of Public Health, POB 2997, Yaoundé, Cameroon; 2 Centre de Recherche en Santé de Nouna, POB 02, Nouna, Burkina Faso; 3 Department of Tropical Hygiene and Public Health, Ruprecht-Karls-University, Im Neuenheimer Feld 324, 69120 Heidelberg, Germany

## Abstract

**Background:**

Pregnant women are a major risk group for malaria in endemic areas. Only little information exists on the compliance of pregnant women with malaria and anaemia preventive drug regimens in the rural areas of sub-Saharan Africa (SSA). In this study, we collected information on malaria and anaemia prevention behaviour in pregnant women of rural Burkina Faso.

**Methods:**

Cross-sectional qualitative and quantitative survey among 225 women of eight villages in rural northwestern Burkina Faso. Four of the villages had a health centre offering antenatal care (ANC) services while the other four were more than five kilometers away from a health centre.

**Results:**

Overall ANC coverage (at least one visit) was 71% (95% in health centre villages vs 50% in remote villages). Malaria and anaemia were considered as the biggest problems during pregnancy in this community. ANC using women were quite satisfied with the quality of services, and compliance with malaria and anaemia prevention regimens (chloroquine and iron/folic acid) was high in this population. Knowledge on the benefit of bed nets and good nutrition was less prominent. Distance, lack of money and ignorance were the main reasons for women to not attend ANC services.

**Conclusions:**

There is an urgent need to improve access of rural SSA women to ANC services, either through increasing the number of rural health centres or establishing functioning outreach services. Moreover, alternative malaria and anaemia prevention programmes such as intermittent preventive treatment with effective antimalarials and the distribution of insecticide-treated bed nets need to become implemented on a large scale.

## Background

Each year between 75.000 and 200.000 infant deaths are attributed to malaria infection in pregnancy globally, and between 200.000 and 500.000 pregnant women develop severe anaemia as a result of malaria in Sub-Saharan Africa (SSA) [1]. Pregnant compared to non-pregnant women are at an increased risk for malaria, and the severity of the clinical manifestations in the women and her foetus depends on the level of pre-pregnancy immunity [2]. While in areas of low malaria endemicity all pregnant women are equally susceptible to the consequences of malaria infection, in areas of high endemicity women appear to be most susceptible during their first pregnancy [3]. However, more recent publications point to significant susceptibility in primigravidae as well as in multigravidae [4].

Pregnancies in women living in malaria endemic regions are associated with a high frequency and density of *P. falciparum *parasitaemia, with high rates of maternal morbidity including fever and severe anaemia, with abortion and stillbirth, and with high rates of placental malaria and consequently low birth weight in newborns caused by both prematurity and intrauterine growth retardation [1,3,5].

In order to reduce malaria-related ill health, regular chemoprophylaxis has been recommended to all pregnant women living in malaria-endemic areas [6]. Most African countries, including Burkina Faso, include routine chemoprophylaxis in their official antenatal care programmes. However, in practice, coverage of chemoprophylaxis is limited due to low accessibility and quality of antenatal care (ANC) services as well as problems with compliance [7]. It has been estimated from a survey in four African countries that less than 20% of women use a prophylactic regimen close to the WHO recommendations [8].

While insecticide-treated bed nets and curtains (ITN) have been shown to substantially reduce malaria morbidity and mortality in children, results from initial trials on the efficacy of ITNs for malaria prevention in pregnancy produced conflicting evidence and were classified as inconclusive by the Cochrane Collaboration [3,9]. However, with the publication of the findings from a major ITN trial in a holoendemic area of western Kenya, the use of ITN during pregnancy is getting more credibility [10].

In Burkina Faso, the official policy for malaria and anaemia prevention in pregnant women comprises of chloroquine and iron/folic acid supplementation respectively. However, in the rural areas of Burkina Faso little information exists on coverage with ANC and on compliance with preventive regimens. We assessed the coverage of antenatal care and investigated the knowledge and adoption of preventive practices with respect to malaria and anaemia in users and non-users of antenatal care.

## Methods

### Study area

The study took place in the rural part of the research zone of the *Centre de Recherche en Santé de Nouna *(CRSN) in Nouna Health District, northwestern Burkina Faso. The CRSN research zone consists of Nouna town and 41 of the surrounding villages with a total population of around 60.000 inhabitants. The Nouna area is a dry orchard savanna, populated mainly by subsistance farmers of different ethnic groups.

Malaria is holoendemic but highly seasonal in this part of Westafrica [11]. Most malaria transmission takes place during or shortly after the rainy season which usually lasts from June until October [11]. Modern health services in the CRSN research zone are limited to four village-based health centres and the district hospital in Nouna town. As a consequence, malaria control is mainly based on home treatment with chloroquine, the official first-line treatment drug in Burkina Faso. Roughly half of all households in the area possess at least one untreated bed net and since 2000, ITN are distributed as part of an ongoing trial in young children [12,13].

The official policy for malaria and anaemia prevention during pregnancy in Burkina Faso consists of a curative dose of 1 500 mg chloroquine during three days followed by a weekly dose of 300 mg chloroquine and a combined dose of daily 200 mg iron and 0.25 mg folic acid. This regimen should be followed from the first ANC visit until six weeks after delivery. Since the year 2002 it is the official policy in Burkina Faso to offer ANC services free of charge. This includes ANC card, physical examination and counselling, and malaria/anaemia prevention drugs. However, urine examination, gloves, and drugs for other concomitant diseases still have to be paid for. Urine examination and gloves usually cost 150–200 F CFA (1 Euro = 650 F CFA).

### Study design

The study was cross-sectional and descriptive in nature, using both qualitative and quantitative methods for data collection. The study was implemented in May and June 2003. The research team comprised of the investigators and six trained local interviewers familiar with the common spoken local languages and French. The questionnaires were pre-tested before administration.

The study took place in eight of the 41 villages of the CRSN study area. Villages were selected as follows: At a first stage, the four villages of the CRSN study area where a health centre exists were purposely selected. To account for the socio-demographic variability and geographical accessibility, in each of these health centre defined sub-areas, another village distant of at least 5 kilometres to the health centre was randomly selected.

### Qualitative research

We conducted six Focus Group Discussions (FGD). Two with pregnant women users of ANC, two with husbands of pregnant women users of ANC, and two with pregnant women non-users of ANC. Respective FGDs were held with groups of six to 12 participants from study villages with and without a health centre.

Key informant interviews were conducted with four maternity health workers, seven traditional birth attendants and 29 women group leaders. The interviews assessed their knowledge, attitudes and practices about malaria and anaemia prevention in pregnancy.

### Quantitative research

The design of the quantitative survey instrument was informed by the results of the qualitative interviews. A structured questionnaire was administered to all women from the eight study villages who had delivered a life child during the last six months (n = 225). Information on births was available through the existing Demographic Surveillance System (DSS) in the study area [14]. The questions focussed on socio-demographic characteristics, obstetrical history, knowledge and practice of preventive measures against malaria and anaemia during pregnancy, factors influencing the utilisation of ANC services and on the compliance with chloroquine and iron/folic acid supplementation during pregnancy. The questionnaires were filled in by the interviewers who also cross-checked given answers with all available ANC cards (n = 156/225).

On the ANC cards, the estimated age of pregnancy at first visit was reported through the fundal height in centimetres, as women were not able to recall their last menstruation period during ANC visits. These data were afterwards transformed into a specific scoring system.

We used two definitions for malaria prophylaxis. A complete curative dose of 1 500 mg chloroquine followed by regularly weekly 300 mg doses afterwards (complete prophylaxis), and an incomplete regimen consisting of only 300 mg weekly doses (incomplete prophylaxis). A combined dose of daily 200 mg iron and 0.25 mg folic acid was defined as a complete prophylaxis.

### Statistical analysis

The data were entered in Microsoft Access 2000, cleaned, and then analysed with Epi Info 2000. Univariate analysis was done with chi-square test or Fisher's exact test to compare proportions for categorical variables. Results were considered to be significant when the 2-sided P value was <0.05.

### Ethical aspects

We received ethical approval from the institutional Ethical Committee at the Department of Tropical Hygiene in Heidelberg, Germany, and the local Ethical Committee in Nouna, Burkina Faso. Oral informed consent was obtained from all participants.

## Results

### Study population

The characteristics of participants on the quantitative survey are shown in table 1. The great majority of the survey women were married, illiterate, housewife/farmers and their ages range between 15 and 49 years. The distribution of ethnicity among survey women was as follows: 41% Bwaba, 39% Marka, 15% Mossi, 3% Samo and 2% others. The median number of pregnancies among survey women was 6 (range 1–13). There were no differences between the background characteristics of users compared to non-users of ANC services, except regarding distance to the nearest ANC services. When comparing women living at a distance ≤ 5 km with women living >5 km, distance was significantly associated with ANC use (p < 0.001).

**Table 1:** Background characteristics of women interviewed

	**ANC**		
**Background Characteristics (%)**	**Users n = 159**	**Non Users n = 66**	**All n = 225**
**Age Group**			
15–19	24 (15)	12 (18)	36 (16)
20–29	81 (51)	37 (56)	118 (52)
30–49	54 (34)	17 (26)	71 (32)
**Education**			
No Schooling	145 (91)	62 (94)	207 (92)
Primary Education	14 (9)	4 (6)	18 (8)
**Parity**			
1	26 (16)	8 (12)	34 (15)
2–3	46 (29)	17 (26)	63 (28)
4–13	87 (55)	41 (62)	128 (57)
Distance*(Km)			
0–4	99 (62)	5 (8)	104 (46)
5–7	43 (27)	59 (89)	102 (45)
8–15	17 (11)	2 (3)	19 (8)

* Distance to the nearest health center in kilometers

### ANC coverage

In this study population, the minimal ANC coverage (defined as at least one ANC visit during pregnancy) was 159/225 (71%) and 63/225 (28%) if we consider the optimal frequency of at least three ANC visits (national goal). Minimal ANC coverage was 97/102 (95%) in villages with a health centre vs 62/123 (50%) in remote villages (p < 0.001), while the optimal ANC coverage was 55/102 (54%) in villages with a health centre vs 8/123 (7%) in remote villages (p < 0.001).

Among ANC users, 27%, 40% and 33% of women visited ANC services one time, two times or more than two times respectively during their pregnancy. The first ANC visit of ANC users was in 14% during the first trimester, in 57% during the second trimester, and in 27% during the third trimester.

### Malaria and anaemia prevention knowledge and behaviour

Malaria and anaemia were considered as the most common diseases during pregnancy by the majority of the participants in the FGD, key informant interviews and survey women. In the Dioula language (lingua franca in the region) malaria is equivalent to "Soumaya", light to moderate anaemia to "Djolidessé" and severe anaemia to "Djoliban". Most women in the FGD were knowledgeable about the malaria prevention effect of chloroquine and the anaemia prevention effect of iron/folic acid.

The knowledge of malaria and anaemia prevention measures by ANC users is given in table 2. Regarding malaria, the majority of survey women stated that it can be prevented with chloroquine (white tablets), while only a minority mentioned mosquito nets or others measures. Regarding anaemia, iron/folic acid (red colour tablets or vitamins) supplementation was stated by the majority of survey women as being protective, while a much smaller percentage of women mentioned nutrition as an important factor. Stating chloroquine and mosquito nets as prevention measures against malaria and iron/folic acid against anaemia was significantly associated with ANC use (p < 0.002; p < 0.001; p < 0.001).

**Table 2:** Knowledge of preventive measures against malaria and anemia

	**ANC**		**All**	***P *Value**
**Knowledge factors (%)**	**Users n = 159**	**Non Users n = 66**	**All n = 225**	**p-value**
**Malaria prevention**				
Chloroquine	128 (66)	35 (59)	163 (65)	<0.001
Mosquito nets	40 (21)	2 (3)	42 (17)	< 0.001
Hygiene	6 (3)	3 (5)	9 (4)	n.s.
Protective clothing	12 (6)	0 (0)	12 (5)	<0.05
Does not know	7 (4)	19 (32)	26 (10)	<0.001
**Anemia prevention**				
Iron/Folic acid	129 (76)	16 (25)	145 (63)	<0.001
Adequate nutrition	29 (17)	8 (13)	37 (16)	n.s.
Does not know	11 (7)	39 (62)	50 (22)	<0.001

Table 3 shows data on self-reported use of malaria and anaemia prophylaxis with chloroquine and iron/folic acid in the population of ANC users together with respective data taken from their ANC cards. A correct prescription of chloroquine (complete prophylaxis) on ANC cards was seen in 60%, and a correct prescription of iron/folic acid (complete prophylaxis) was seen in 78%. In contrast, self-reported oral information during ANC visits on complete and incomplete prophylaxis regimens were lower and matched well with self-reported information on the chloroquine and iron/folic acid dosages taken. Most women reported being compliant with the oral information on chloroquine and iron/folic acid regimens from first ANC visit until delivery.

**Table 3:** Prescription in ANC card of chloroquine prophylaxis and iron/folic acid supplemen-tation, self-reported ANC instructions and self-reported intake among ANC users (n = 159)

	**Chloroquine (%)**	**Iron/folic acid (%)**
**Prescription on ANC card**		
Complete prophylaxis	95 (60)	124 (78)
Incomplete prophylaxis	29 (18)	-
Incorrect prescription	32 (20)	32 (20)
No prescription (no ANC card)	3 (2)	3 (2)
**Instructions given at ANC visits (self-reported)**		
Complete prophylaxis explained	46 (29)	111 (70)
Incomplete prophylaxis explained	65 (41)	-
Incorrect instructions given	41 (26)	41 (26)
No instructions given	7 (4)	7 (4)
**Dosages taken (self-reported)**		
Complete prophylaxis	46 (29)	149 (94)
Incomplete prophylaxis	67 (42)	-
Incorrect dose	43 (27)	8 (5)
No prophylaxis	3 (2)	2 (1)
**Duration of chloroquine and iron/folic acid prophylaxis**		
From 1^st ^ANC until before delivery	20 (13)	20 (13)
From 1^st ^ANC until delivery	115 (72)	124 (78)
From 1^st ^ANC until after delivery	21 (13)	13 (8)
No prophylaxis	3 (2)	2 (1)

### Factors influencing the use of ANC services

Of ANC users, the great majority reported to be satisfied with the quality of ANC services. Only 16% of ANC users were aware of the fact that ANC services have recently become free of charge, and 42% reported that they still had paid for services. Most (73%) ANC users had paid between zero and 200 F CFA, while 12% and 6% had paid between 200 and 1.500 and between 1.500 and 7.500 F CFA respectively.

Apart from distance to the next health centre, lack of resources and ignorance were the most frequent stated reasons during qualitative and quantitative interviews why women did not attend ANC services. The majority of ANC non-users reported no specific prophylaxis during their pregnancy, but 6% took irregularly self medication for malaria/anaemia prevention. Moreover, 30% of ANC non-users had sought advice from traditional birth attendants (TBA).

Typical statements in the FGDs with non-users of ANC services werecollected as follow:

• *"ANC is not free of charge, you must pay for ANC card and the medicine and it could be up to 500 CFA and for me I find it is expensive. Our husbands find it expensive too, that is why the majority of us can not attend ANC services".*

• *"I don't know the advantages of ANC services, some of the pregnant women if they don't fell sick they wouldn't accept to attend ANC ".*

• *"There is lack of advices from health workers, if you go to the ANC for the 1*^st^*time, they give drugs, they don't tell you when you should come back and how many visits you should attend. That is how they do here".*

• *"If you are not sick, you don't pay for drugs, what you pay for is the ANC card at 150 F CFA...."*

• *" But even the ANC card is free of charge, only the gloves cost 100 F CFA...."*

• *"I had not yet heard about the free ANC services ... Since I know now that ANC is free of charge I will attend the services."*

• *"We do not have money to go to the health centre it will be nice to have one in our village".*

Itching, vomiting and fatigue were the most frequently stated side effects of malaria/anaemia prevention drug regimens during interviews, sometimes leading to non-compliance. Most women also stated that bed nets are considered too expensive for their household.

## Discussion

The main findings of this study are (1) that coverage of antenatal care is far from complete, particularly in villages without a health centre, (2) that malaria- and anaemia-related knowledge and compliance with preventive measures is comparatively high with a wide gap between users and non-users of antenatal care and (3) that health services need to improve their response to the women's need for preventive care in pregnancy.

### Use and coverage of antenatal care

In this community-based study from a rural area of Burkina Faso, we found an overall ANC coverage of 71%, which is however not representative given the used methodology. Most women had two ANC visits during their pregnancy, mainly during the second and third trimester. As we found ANC coverage to be much higher in villages with a health centre compared to villages quite distant from a health centre, and as we included similar numbers of health centre and non-health centre villages, our ANC coverage figure is likely to be an overestimate. The national demographic and health survey in Burkina Faso claims an ANC attendance (at least one ANC during one pregnancy) of 59% [15]. Although health workers from rural health centres in Burkina Faso are advised to do regularly outreach work in the villages of their respective catchment areas, in practice such visits are rare due to a number of reasons such as lack of transport. Our findings thus support the need for better access of rural SSA women to ANC services [16].

Other reasons given for non-use of ANC services included ignorance and lack of money. This confirms similar observations from other rural African areas [20]. Interestingly, the Burkinabé Government had recently changed its policy towards free ANC service provision. However, our findings show that this policy is rather confusing as some parts of ANC procedures are not included. Consequently it was not yet fully understood by the population. However, this change in policy was considered promising during our interviews, and it was also reassuring that most of the ANC users were satisfied with the quality of ANC services.

### Knowledge and compliance with preventive interventions related to malaria and anaemia

Malaria and anaemia were seen as important disease entities during pregnancy in our interviews. Moreover, most women interviewed were quite knowledgeable about effective malaria/anaemia prevention measures. However, compared to ANC users ANC non-users were significantly less knowledgeable about malaria/anaemia prevention measures. The interpretation is not straight forward because more knowledgeable women may be more likely to attend antenatal care or increased knowledge may be the result of health education in anatenatal care; most likely both effects contribute to the observed gap between ANC users and ANC non-users.

### Responsiveness of health services

There is now a broad agreement on the need of new strategies for community participation on the implementation of effective malaria control activities, as well as for a better education of service providers of both the public and the private sector [17].

It was reassuring to find nearly all of the women with reported ANC use to have an ANC card in their house. Specific prescriptions were found on most cards, but in particular malaria prophylaxis prescriptions were often not complete. This explains why self-reported compliance with recommended prevention regimens was sub-optimal with regard to malaria. Such discrepancies have been observed already in a recent study in pregnant women of Nouna town [18]. However, self-reported compliance matched well with the reported oral instructions given by respective health workers. This points to the importance of correct oral information in rural areas with high prevalence of illiteracy [19].

The best model for effective and cost-effective malaria prevention during pregnancy in SSA still needs to be developed. Chloroquine has been the mainstay for malaria control in sub-Saharan Africa (SSA), but the emergence of chloroquine-resistant *Plasmodium falciparum *has put into question the efficacy of this well-known drug [21]. The first cases of in vitro and in vivo Chloroquine resistance in Burkina Faso were seen in 1983 and 1988, respectively, and reported clinical failure rates after use of Chloroquine for treatment of uncomplicated malaria in children were around 5% in the early 1990s and 10% during the most recently performed surveys [22]. Although this is considered still below the threshold of clinical failures considered to require a change of first-line treatment, it has recently been shown that chloroquine failed to prevent malaria in pregnant women of Burkina Faso [23]. Current alternatives include intermittent treatment with pyrimethamine-sulfadoxine and the use of ITNs. Sulphadoxine-pyrimethamine, given in 1–3 therapeutic dosages during the second and third trimester of pregnancy, has recently been demonstrated to be an effective and cost-effective schedule for malaria prevention [24]. Compared to chloroquine prophylaxis, this regimen has the advantage that it is given to women when they attend antenatal clinics, thus avoiding problems with compliance. ITNs are increasingly considered as an important tool in the prevention of malaria in young children and pregnant women, and the provision of ITN through ANC services has recently been proposed as a promising distribution channel [13,25]. The findings of this study confirm the cost barrier to the private purchase of bed nets and ITNs and thus support the call for major subsidies if a high ITN coverage is going to be achieved [12,13,25].

## Competing interests

None declared.

## Authors'Contributions

CM, AJ and OM designed the study. FS and BK were responsible for the conduct of the study in Burkina Faso. CM analysed the data. All authors contributed to the interpretation of the data, helped write the paper, and read and approved the final manuscript.

## Pre-publication history

The pre-publication history for this paper can be accessed here:


